# Perampanel use in pediatric autoimmune encephalitis-related seizures and epilepsy: a retrospective case series

**DOI:** 10.3389/fneur.2026.1773184

**Published:** 2026-05-26

**Authors:** Jiaqin Yi, Qing Lu, Ling Hu, Jiehui Ma, Qiaoqiao Qian, Dan Sun

**Affiliations:** Department of Neurology, Wuhan Children’s Hospital of Tongji Medical College, Wuhan, China

**Keywords:** autoimmune encephalitis, children, epilepsy, perampanel, seizures

## Abstract

**Objective:**

There is limited data regarding the use of antiseizure medications (ASMs) for autoimmune encephalitis (AE)-related seizures and epilepsy, particularly in pediatric patients. Perampanel (PER) was associated with seizure improvement in controlling seizures of inflammatory etiology. The objective of this study was to describe seizure outcomes and tolerability after PER administration in pediatric patients with AE-related seizures and epilepsy.

**Methods:**

This retrospective, single-center study (2020–2025) reviewed 14 pediatric patients diagnosed with AE who received PER during either the acute symptomatic phase or the chronic phase of autoimmune-associated epilepsy. Clinical data, seizure outcomes, concomitant treatments, and adverse events were retrospectively collected and descriptively analyzed.

**Results:**

Fourteen pediatric patients with AE (9 male, 5 female; median age 8.5 years) were treated with PER. The median maintenance dose of PER ranged from 2 to 4 mg per day. According to the classification by the International League Against Epilepsy (ILAE), the patients were divided into two groups: (a) acute symptomatic seizures secondary to AE (ASSAE), and (b) autoimmune-associated epilepsy (AAE). In the ASSAE group, 42.8% (3/7) of the patients achieved seizure resolution during the acute phase. In the AAE group, at the 3-month endpoint, 57.1% (4/7) of patients achieved a ≥ 50% reduction in seizure frequency compared to baseline, meeting the responder criteria. Additionally, 42.8% (3/7) of patients in the AAE group achieved seizure freedom at the 3-month endpoint. No serious adverse events were reported in either group.

**Conclusion:**

In this small retrospective case series, seizure improvement was observed in some pediatric patients with AE-related seizures or epilepsy after PER administration, without serious adverse events. Because most patients received concomitant ASMs and immunotherapy, these findings should be interpreted as preliminary and hypothesis-generating.

**Clinical trial registration:**

https://www.chictr.org.cn/showproj.html?proj=191349, identifier: ChiCTR2300074696.

## 1 Introduction

Autoimmune encephalitis (AE) is an inflammatory neurological disorder that predominantly affects children and adolescents (1). The pathogenesis of AE is associated with antibodies targeting neuronal cell-surface antigens—such as the N-methyl-D-aspartate receptor (NMDAR), leucine-rich glioma-inactivated 1 (LGI1), contactin-associated protein-like 2 (CASPR2), *α*-amino-3-hydroxy-5-methyl-4-isoxazole propionic acid receptor (AMPAR), and *γ*-aminobutyric acid B receptor (GABABR)—or synaptic proteins, including glutamic acid decarboxylase 65 (GAD65) and neurexin-3α (2). However, some patients who present with the clinical phenotype of AE test negative for known autoantibodies; these cases are classified as antibody-negative autoimmune encephalitis.

The main clinical manifestations of pediatric AE include seizures, movement disorders, and psychiatric or behavioral symptoms (3). During the acute stage, patients may develop status epilepticus (SE), refractory status epilepticus (RSE), or even super-refractory status epilepticus (SRSE). In terms of long-term outcomes, chronic epilepsy develops in approximately 3.1% of patients with AE (4). The International League against Epilepsy (ILAE) has recommended the term “autoimmune-associated epilepsy” (AAE) to describe chronic seizures secondary to autoimmune brain diseases (5). Therefore, selecting appropriate antiseizure medications (ASMs) is critical for achieving seizure control in both the acute and chronic phases of AE.

The *α*-amino-3-hydroxy-5-methyl-4-isoxazole propionate receptors (AMPARs), a subtype of ionotropic glutamate receptors (iGluRs), are involved in rapid excitatory synaptic transmission and synaptic plasticity (6). AMPAR-mediated excitotoxicity has been implicated in the inflammatory processes of autoimmune encephalomyelitis models and in epileptogenic mechanisms (7). Perampanel (PER), a selective non-competitive AMPAR antagonist, has demonstrated broad-spectrum antiepileptic activity across various seizure types (8). Recent evidence supports the efficacy of PER in controlling seizures with suspected inflammatory etiologies (9–11). However, clinical data on the use of PER for AE-related seizures and epilepsy, particularly in pediatric populations, remain limited. Therefore, this study was aimed to evaluate the efficacy of PER in the treatment of seizures associated with AE in pediatric patients.

## 2 Methods

### 2.1 Study population

This retrospective study was conducted in the Department of Pediatric Neurology at Wuhan Children’s Hospital from December 2020 to August 2025. Pediatric patients aged 0–18 years who were clinically diagnosed with AE and treated with perampanel (PER) were included.

According to the International League against Epilepsy (ILAE) Autoimmune and Inflammation Working Taskforce classification, the enrolled patients were divided into two groups: (a) acute symptomatic seizures secondary to autoimmune encephalitis (ASSAE), and (b) autoimmune-associated epilepsy (AAE), which was defined as recurrent unprovoked seizures persisting after adequate immunotherapy and in the absence of evidence of active encephalitis (5). In this study, adequate immunotherapy referred to completion of standard first-line immunotherapy, with additional second-line or targeted immunotherapy when clinically indicated. Absence of active inflammation was assessed based on clinical stabilization, no progressive encephalopathic symptoms, improvement or stabilization of behavioral and cognitive manifestations, absence of new inflammatory CSF abnormalities, and no new or progressive inflammatory lesions on brain MRI. ASSAE were defined as seizures occurring during the active inflammatory phase of AE, including seizures at disease onset or during the acute/subacute encephalitic stage before clinical stabilization. This definition was not restricted to seizures occurring within 7 days after AE symptom onset, because the active inflammatory phase of AE may extend beyond the conventional 7-day window used for some structural brain insults. The interval from AE symptom onset to first seizure onset and the interval from seizure onset to PER initiation were recorded separately. Exclusion criteria included incomplete medical records and poor treatment adherence. Written informed consent was obtained from the parents or legal guardians of all participants.

All patients underwent a comprehensive diagnostic work-up, including blood tests, cerebrospinal fluid (CSF) analysis, brain magnetic resonance imaging (MRI), and electroencephalography (EEG). Antibody testing for NMDAR, AMPAR subunit 2, LGI1, CASPR2, and GABABR was performed on both serum and CSF samples. Tissue-based assays (TBA) and cell-based assays (CBA) were conducted following standard procedures (Kindstar Global, China). The study protocol was approved by the Ethics Committee of Wuhan Children’s Hospital (ID number: 2021R133-E02). The study has been registered in the Chinese Clinical Trial Registry (ChiCTR) (Registration No.: ChiCTR2300074696).

### 2.2 Dosage regimen and data collection

Perampanel (2 mg per tablet; Fycompa, Eisai Co.) was administered orally once daily to pediatric patients diagnosed with AE. Treatment began with an initial dose of 1–2 mg/day before bedtime. In the absence of adverse events or tolerability issues, the dose was gradually titrated according to seizure severity, body weight, clinical urgency, and physician judgment. Although titration up to 8 mg/day within approximately 2 weeks was permitted in selected severe cases, most patients received maintenance doses of 2–4 mg/day. This individualized approach reflected the need for rapid seizure control in some patients with refractory seizures or status epilepticus, while allowing dose reduction or slower titration in patients with behavioral, psychiatric, or tolerability concerns. Down-titration was allowed in cases of intolerable adverse effects. Clinical data collected included age, sex, prodromal symptoms, intellectual development, seizure type, intensive care unit (ICU) admission, modified Rankin Scale (mRS) score, prior antiseizure medications (ASMs), immunotherapy details, time to seizure onset, and timing of PER initiation.

### 2.3 Efficacy and safety assessment

The primary outcome was seizure outcome after PER administration. For the ASSAE group, acute seizure cessation after PER administration was defined as meeting both of the following criteria: (a) PER was the last ASM introduced before seizure cessation, without modification of concomitant ASMs during the 72-h assessment period; and (b) no clinically observed seizures occurred within 72 h after PER initiation. Seizure cessation was assessed based on continuous bedside clinical observation and EEG monitoring when available. EEG was performed during hospitalization in all ASSAE patients; however, continuous EEG monitoring was not uniformly available throughout the entire 72-h post-PER assessment window. Therefore, subclinical seizures could not be completely excluded in all patients (12). For the AAE group, baseline seizure frequency was recorded over a 28-day period before PER initiation. Patients who achieved a ≥ 50% reduction in seizure frequency after 3 months of treatment were classified as responders, while seizure freedom was defined as complete seizure control during the final three-month evaluation period.

The secondary outcome included assessment of PER retention rate during follow-up. Functional outcomes were evaluated using the modified Rankin Scale (mRS, scores 0–6). A good outcome was defined as mRS 0–2, whereas a poor outcome was defined as mRS > 3. Functional decline was defined as an increase of more than one point in mRS from baseline to the most recent follow-up (13). Behavioral, mood, and memory changes were assessed retrospectively based on neurological examination, caregiver reports, and documentation in the medical records. Standardized neuropsychological scales were not routinely available for all patients. All adverse events were recorded and assessed from the first PER administration until the last follow-up. Follow-up duration was defined as the interval from PER initiation to the last available clinical assessment. In the AAE group, PER exposure and follow-up were longer because PER was used for chronic epilepsy management.

### 2.4 Statistical analysis

Given the retrospective case-series design and small sample size, no inferential statistical tests were performed. The analysis was descriptive and exploratory. Continuous variables were expressed as mean ± standard deviation (SD) or median and interquartile range (IQR), as appropriate. Categorical variables were summarized as counts and percentages. Patients with incomplete medical records were excluded, and no imputation was performed for missing data. All statistical analyses were performed using SPSS Statistics version 26.0 (IBM Corp., Armonk, NY, United States).

## 3 Results

### 3.1 Study flow

A total of 16 pediatric patients with AE were screened at Wuhan Children’s Hospital between December 2020 and August 2025. Fourteen patients met all inclusion criteria and were included in the final analysis. Of these, 9 (64.3%) were male and 5 (35.7%) were female, with a median onset age of 8.5 years (range: 5.3–10.3 years). Seven patients were classified as having acute symptomatic seizures secondary to ASSAE (patients 3, 7, 10, 11, 12, 13, and 14), while the remaining seven were categorized as having AAE (patients 1, 2, 4, 5, 6, 8, and 9).

### 3.2 Treatment with perampanel in the ASSAE group

#### 3.2.1 Baseline characteristics of ASSE group

**Figure 1:**
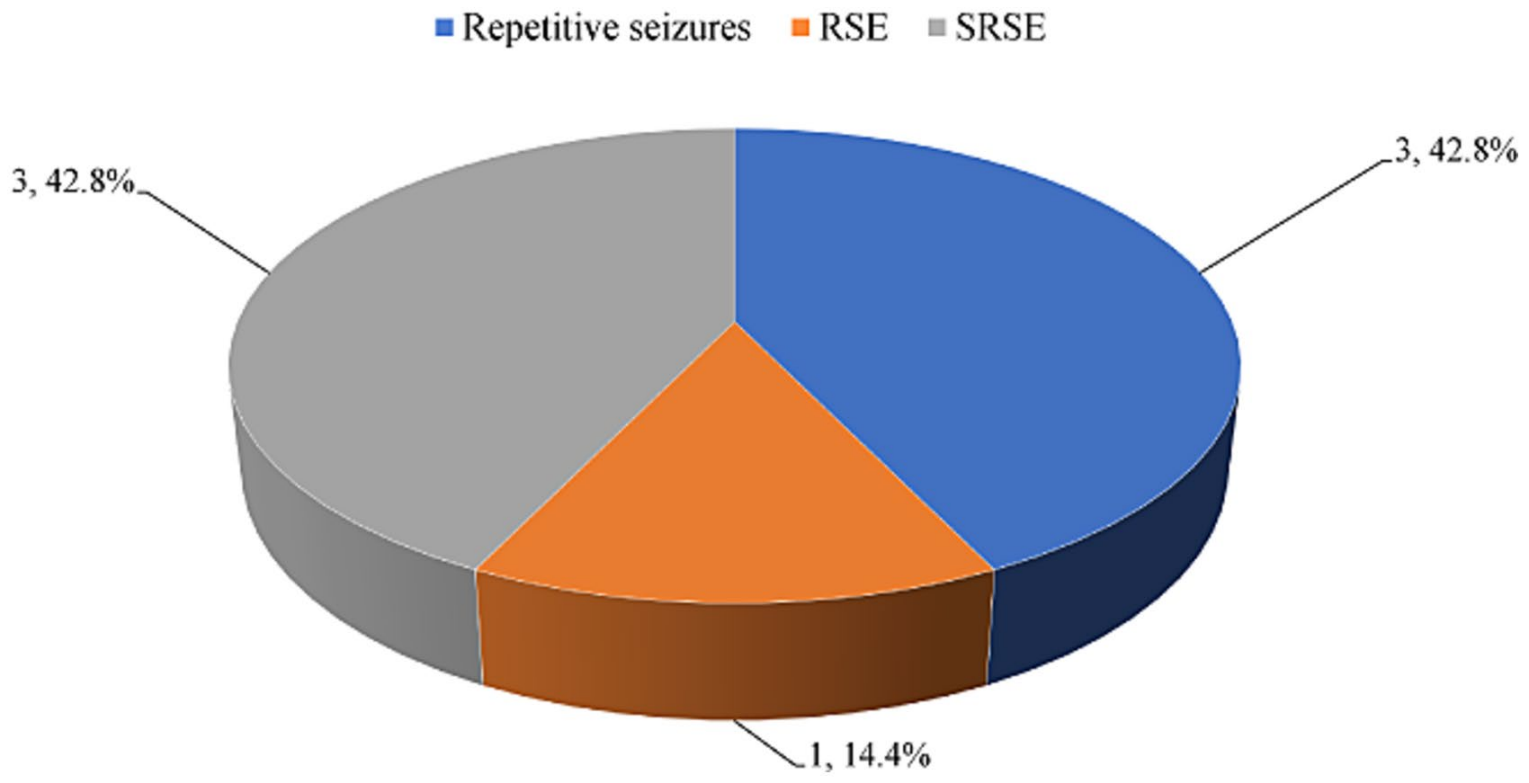
Seizure characteristics in children with acute symptomatic seizures secondary to autoimmune encephalitis (ASSE). Proportion of children experiencing repetitive seizures, refractory status epilepticus (RSE), or super-refractory status epilepticus (SRSE).

The ASSAE group included 3 males and 4 females, with a mean onset age of 8.8 ± 3.3 years. All children exhibited prodromal symptoms, including respiratory infections (*n* = 4) or acute gastroenteritis (*n* = 3). Four children were diagnosed with antibody-negative AE, while three had anti-N-methyl-D-aspartate receptor (NMDAR) encephalitis (1). Initial clinical manifestations included seizures (*n* = 3), psychiatric symptoms (*n* = 1), sleep disturbances (*n* = 1), dyskinesia (*n* = 1), and decreased consciousness (*n* = 1). According to the 2017 ILAE classification, seizure types comprised generalized tonic–clonic seizures in 2 patients (28.6%) and focal motor seizures in 5 patients (71.4%) (14). Based on seizure type, duration, and drug responsiveness, 3 patients (42.8%) had repetitive seizures, 1 (14.4%) had refractory status epilepticus (RSE), and 3 (42.8%) developed super-refractory status epilepticus (SRSE) (15) (Figure 1). Five patients (71.4%) required intensive care unit (ICU) admission due to disease severity. All seven patients presented with severe functional impairment, with modified Rankin Scale (mRS) scores ≥ 3 at onset (Table 1).

**Table 1:** Clinical features of ASSAE group treated with perampanel.

Patient	Age at AE onset (year)/gender	Seizure type	AE etiology	ICUs admission	Previous ASMs	Immunotherapy ongoing at PER initiation	Interval from seizure onset to PER initiation	Duration of ongoing seizure before PER treatment	Responder	Change in mRS from baseline e
Nr3	10.1/male	GTCS	Negative autoimmnue encephalitis	Yes	LEV	IVIG+ IVMP	96 h	1-7d	None	2
Nr7	12.0/female	Focal motor	Anti-NMDAR-encephalitis	Yes	LEV, LCM, TPM	IVIG+ IVMP+RTX	1,080 h	>7d	Yes	4
Nr10	11.0/female	Focal motor	Anti-NMDAR-encephalitis	No	LEV	IVIG+ IVMP+RTX	168 h	>7d	Yes	3
Nr11	3.2/female	Focal motor	Negative autoimmnue encephalitis	Yes	LEV, LCM, TPM	IVIG+ IVMP+TCZ	720 h	>7d	None	2
Nr12	9.1/male	Focal motor	Negative autoimmnue encephalitis	Yes	VPA, ZNS, NZP	IVIG+ IVMP+TCZ	1,392 h	>7d	None	3
Nr13	11.0/female	Focal motor	Anti-NMDAR-encephalitis	No	None	IVIG+ IVMP	72 h	1-7d	Yes	2
Nr14	5.3/male	GTCS	Negative autoimmnue encephalitis	Yes	None	IVIG+ IVMP+RTX	120 h	>7d	None	3

AE, Autoimmnue encephalitis; ASM, Antiseizure medication; LCM, Lacosamide; LEV, Levetiracetam; IVIG, IV immunoglobulins; IVMP, IV methylprednisolone; RTX, Rituximab; GTCS, Generalized tonic–clonic seizure; ICU, Intensive care unit; TPM, Topiramate; VPA, Valproate acid; NZP, Nitrazepam; PER, Perampanel; ZNS, Zonisamide.

#### 3.2.2 Primary outcome of ASSE group

PER was not necessarily initiated at seizure onset. In most ASSAE patients, PER was introduced after other ASMs had already been administered. Specifically, PER was administered as the fourth ASM in 3 of 7 patients (42.8%), as the second ASM in 2 patients (28.6%), and as monotherapy in 2 patients (28.6%). The initial dose was 1 mg/day, and the median maintenance dose was 3 mg/day (range: 2–4 mg/day). All patients in the ASSAE group received immunotherapy during the acute phase. First-line immunotherapy consisted of intravenous immunoglobulin (IVIG) and intravenous methylprednisolone (IVMP) in all seven patients. Additional second-line or targeted immunotherapy was administered according to clinical severity and treatment response, including rituximab in three patients and tocilizumab in two patients. PER was initiated while immunotherapy was ongoing in all ASSAE patients. Therefore, seizure outcomes in this group should be interpreted in the context of concomitant immunotherapy and other ASMs. Seizure cessation, confirmed by both clinical and EEG evaluation, was achieved in 3 of 7 children (42.8%). All responders were diagnosed with anti-NMDAR encephalitis. The median time to seizure resolution following PER initiation was 56 h (range: 48–72 h). Two patients (patients 7 and 12) with SRSE, who had received three ASMs before PER, achieved seizure control within 48 h. Two others (patients 3 and 10) received PER as first-line adjunctive therapy, while patients 13 and 14 received PER monotherapy; patient 13 achieved seizure freedom within 72 h. The interval from seizure onset to initial PER administration ranged from 108 to 1,392 h (median: 168 h). Among the non-responders (patients 3, 11, 12, and 14), PER contributed to partial seizure control, although it was not the final agent to achieve cessation per predefined criteria. For example, in patient 3, seizure freedom was achieved after the addition of clobazam as a third ASM. Patients 11, 12, 13, and 14 demonstrated partial seizure reduction during PER treatment.

#### 3.2.3 Secondary outcome of ASSE group

At the last follow-up (median = 8 months; range = 6–12 months), all patients achieved favorable functional outcomes, with 4 patients scoring mRS 1 and 3 patients scoring mRS 0. The median mRS score improved by 3 points from baseline. No patient discontinued PER during follow-up. One patient (patient 11) experienced mild aggression, which was tolerated without the need for dose adjustment.

### 3.3 Treatment with perampanel in the AAE group

#### 3.3.1 Baseline characteristics of AAE group

Seven children (6 males, 1 female) were included in the AAE group, all with seronegative autoimmune encephalitis. The mean age at PER initiation was 7.3 ± 2.3 years, and the mean epilepsy duration was 8.3 ± 1.3 months. All patients experienced focal seizures; patient 6 also had spasms, and patient 9 presented with additional generalized tonic–clonic seizures. Following adequate immunotherapy, 6 of 7 patients (85.7%) showed significant improvement in memory and behavior. However, patient 6 exhibited persistent deficits in memory, calculation, and perception. Before PER initiation, all patients in the AAE group had failed a mean of three ASMs, indicating that this group largely represented drug-resistant or difficult-to-treat epilepsy at the time PER was introduced. The prior ASMs included levetiracetam, oxcarbazepine, lacosamide, nitrazepam, clonazepam, valproic acid, topiramate, and phenobarbital (Table 2).

**Table 2:** Clinical features of AAE group treated with perampanel.

Patient	Age at AE onset (year)/gender	Seizure type	Monthly baseline seizure frequecy	AE etiology	Previous ASMs	Immunotherapy	Interval from epilepsy onset to PER initiation (month)	Responder
Nr1	4.9/male	Focal motor	12	Negative autoimmnue encephalitis	LEV, VPA, OXC, CZP, PB	IVIG+ IVMP	31.5	Yes
Nr2	5.1/female	Focal motor	305	Negative autoimmnue encephalitis	LCM, LEV	IVIG+ IVMP+MMF	32	None
Nr4	9.8/male	Focal motor	616	Negative autoimmnue encephalitis	LEV, OXC	IVIG+ IVMP+TCZ	35	Yes
Nr5	7.7/male	Focal motor	14	Negative autoimmnue encephalitis	LEV, OXC	IVIG+ IVMP	32	Yes
Nr6	5.5/male	Focal motor and spasm	102	Negative autoimmnue encephalitis	NZP, TPM, VPA, LCM	IVIG+ IVMP+TCZ	33	None
Nr8	8.0/male	Focal motor	13	Negative autoimmnue encephalitis	LEV, CZP	IVIG+ IVMP	31	Yes
Nr9	10.13/male	Focal motor and GTCS	621	Negative autoimmnue encephalitis	VPA, LEV, OXC, PB	IVIG+ IVMP+RTX	32	None

AE, Autoimmnue encephalitis; ASM, Antiseizure medication; LCM, Lacosamide; LEV, Levetiracetam; IVIG, IV immunoglobulins; IVMP, IV methylprednisolone; RTX, Rituximab; GTCS, Generalized tonic–clonic seizure; TPM, Topiramate; VPA, Valproate acid; NZP, Nitrazepam; MMF, Mycophenolate mofetil; CZP, Clonazepam; OXC, Oxcarbazepine; PB, Phenobarbital; PER Perampanel; TCZ, Tocilizumab.

#### 3.3.2 Primary outcome of AAE group

The mean initial and maintenance doses of PER were 1 mg/day and 4 mg/day, respectively. The median baseline seizure frequency was 102 episodes (range: 13.5–460.5) per 28 days. The most commonly used concomitant ASMs were levetiracetam (85.7%, *n* = 6) and oxcarbazepine (57.1%, *n* = 4). After 3 months of treatment, 4 of 7 patients (57.1%) achieved ≥50% seizure reduction from baseline (responder rate), and 3 of 7 patients (42.8%) achieved seizure freedom. We also explored concomitant ASM patterns descriptively. Because of the small cohort size, no formal association between specific ASM combinations and PER response could be determined. However, nonresponders tended to have higher baseline seizure frequencies and more frequent exposure to multiple prior or concomitant ASMs, including oxcarbazepine and phenobarbital in some cases, which may have influenced PER exposure and clinical response.

#### 3.3.3 Secondary outcome of AAE group

The mean duration of PER exposure was 16.6 ± 6.4 months, with a retention rate of 85.7% (6/7) at the last follow-up. One patient (patient 9) discontinued treatment due to insufficient therapeutic response. No serious adverse events were observed. The most common side effects were dizziness (28.6%, 2/7) and irritability (14.3%, 1/7), which generally occurred during the initial titration period.

## 4 Discussion

Studies evaluating the efficacy of ASMs in AE remain limited (16, 17). In this retrospective case series, seizure improvement was observed in some pediatric patients with AE-related seizures or epilepsy after PER administration. Given the small sample size, lack of a control group, and frequent use of concomitant ASMs and immunotherapy, our findings should be interpreted as preliminary observations rather than definitive evidence of PER efficacy. Our findings indicate that PER may be beneficial in the treatment of active neuroinflammation (acute symptomatic seizures secondary to AE, ASSE) as well as in ongoing autoimmune processes.

In the central nervous system (CNS), glutamate serves as the principal excitatory neurotransmitter. Its effects are mediated by a family of glutamate receptors (GluRs), including ionotropic (iGluRs) and metabotropic (mGluRs) subtypes. These receptors are expressed by most neural and immune cells, playing critical roles in regulating neuroinflammation (18). Among iGluRs, AMPA and NMDA receptors are particularly important in seizure pathogenesis due to their regulation of ion permeability, especially Ca^2+^ influx (19). Excessive Ca^2+^ entry through Ca^2+^-permeable AMPA receptors (CP-AMPARs) contributes to excitotoxicity in several neurological disorders. Thus, modulation of CP-AMPARs represents a potential therapeutic strategy for seizure termination (20). Preliminary evidence suggests that early administration of AMPA receptor antagonists yields promising results in seizure control (21, 22).

The classification of neural autoantibodies is crucial for AE prognosis. These antibodies target either neural cell-surface antigens—such as NMDAR, AMPAR subunit 2, LGI1, CASPR2, and GABABR—or intracellular antigens including GAD65 and Ma2. Patients with cell-surface antibodies generally exhibit better responses to immunotherapy and ASMs compared with those with intracellular antibodies, who face higher risks of AAE (2). However, certain patients with surface antibody–mediated AE remain resistant to ASMs (23). Managing seizures in these cases is challenging, underscoring the need for targeted pharmacologic approaches. Recent reports have described favorable outcomes with PER in anti-AMPAR and anti-NMDAR encephalitis, suggesting that its antiseizure effects may involve modulation of receptor activity and attenuation of neuronal hyperexcitability (9, 10). These findings warrant further investigation into PER’s role in AE-related epileptogenesis.

Accumulating evidence implicates neuroinflammation as a key mechanism in epileptogenesis. Proinflammatory cytokines (PICs)—including tumor necrosis factor-*α* (TNF-α), interleukin-1β (IL-1β), and interleukin-6 (IL-6)—promote neuronal hyperexcitability and blood–brain barrier dysfunction (24). IL-6 facilitates the differentiation of T cells into Th17 subsets, while IL-17 recruits immune cells such as microglia and astrocytes to sites of inflammation (25). Therefore, drugs targeting inflammatory pathways may reduce the risk and severity of epilepsy. Preclinical data have shown that PER exerts anti-inflammatory effects by suppressing PIC release and modulating apoptotic pathways, further supporting its therapeutic potential in AE-related seizures (26).

In our cohort of 14 pediatric AE patients treated with PER (7 with ASSE and 7 with AAE), PER at a maintenance dose of 1–4 mg/day demonstrated favorable outcomes. In the ASSE group, 42.8% (3/7) achieved seizure resolution. Previous research in refractory or super-refractory status epilepticus (RSE/SRSE) reported a 33.3% (27/81) response rate to PER, with better outcomes when PER was introduced earlier and with fewer concomitant ASMs (22). Our findings were consistent: responders had fewer concurrent ASMs (<3) compared to nonresponders (>3). Moreover, prior studies indicated that patients with nonconvulsive status epilepticus respond better than those with focal motor or generalized tonic–clonic seizures (22). Although our sample size was limited, no efficacy differences were observed between seizure types, and larger studies are needed to clarify these associations.

Evidence regarding seizure outcomes in seronegative AE remains inconsistent. Some studies have reported favorable outcomes in seronegative AE (27), whereas others suggest that seronegative patients may have poorer seizure outcomes, greater diagnostic uncertainty, or delayed treatment optimization (28). In our cohort, all patients who developed AAE were antibody-negative, and seronegative patients in the ASSAE group were also less likely to meet the predefined responder criteria. This observation may reflect selection bias, small sample size, delayed diagnosis, unrecognized antibodies, or a distinct immune-mediated epileptogenic process (16). Nevertheless, it suggests that children with seronegative AE and persistent seizures may benefit from more aggressive diagnostic reassessment, including repeated antibody testing when appropriate, careful review of CSF, MRI, and EEG findings, and early optimization of immunotherapy and ASM strategies. This finding should be interpreted as hypothesis-generating and requires validation in larger cohorts.

The etiology of AAE may involve post-encephalitic structural injury, residual network hyperexcitability, or persistent immune-mediated epileptogenic mechanisms after the clinically active inflammatory phase has subsided (5). This concept differs from active encephalitis, which was excluded in our AAE definition. T-cell–mediated inflammation may induce long-term neuronal network alterations that promote chronic seizures (29). Conventional immunotherapy is typically less effective in these patients (5). Sodium channel blockers have shown some benefit, but evidence on other ASMs remains sparse (29). In our AAE cohort, all patients were seronegative, and PER was administered after a median of 2.7 prior ASMs. The 50% responder rate was 57.1% (4/7). PER appeared ineffective for epileptic spasms, as observed in one refractory case, but showed good control of focal motor seizures. Responders also tended to have lower baseline seizure frequencies, though the small sample limits generalization.

It should be emphasized that many patients, particularly in the AAE group, had already received multiple ASMs before PER initiation. Therefore, the cohort should be regarded as a difficult-to-treat population rather than an unselected AE cohort. Prior and concomitant ASM exposure may have influenced both seizure outcomes and adverse-event profiles, and limits any causal attribution of seizure improvement to PER alone. Drug–drug interactions should also be considered when interpreting PER response. Enzyme-inducing ASMs, such as carbamazepine, oxcarbazepine, phenytoin, and phenobarbital, may reduce PER exposure and potentially attenuate treatment response (8). In our small cohort, several nonresponders had received multiple ASMs, including oxcarbazepine or phenobarbital. However, because of the limited sample size, no reliable association between ASM combinations and PER response could be established.

No serious adverse events occurred in either group. The most frequent adverse effects were dizziness, somnolence, and irritability. Given the psychiatric vulnerability of AE patients and the mood-altering potential of ASMs such as brivaracetam, levetiracetam, and topiramate, clinicians should remain vigilant for psychiatric side effects, including suicidal ideation (30). If adverse events emerge, PER dosage reduction or temporary discontinuation is recommended until symptoms resolve.

This study has several limitations. First, as a retrospective, single-center case series with a small sample size, selection bias cannot be excluded. This is particularly relevant because seizure outcomes differed between patients with anti-NMDAR encephalitis and those with seronegative AE. Second, the limited sample size precluded reliable assessment of associations between seizure type, antibody status, ASM combinations, and treatment outcomes, and also increased the risk of type II error. Third, PER dosing and timing were individualized based on clinical judgment, which may have influenced response rates. Fourth, seizure frequency data were partly caregiver-reported and therefore subject to recall bias. Fifth, behavioral, mood, and memory outcomes were assessed retrospectively from clinical documentation rather than standardized neuropsychological testing. Furthermore, because most patients received concomitant ASMs and immunotherapy, the independent contribution of PER cannot be fully disentangled. Prospective controlled studies are needed to validate these preliminary observations. Finally, although EEG was performed during hospitalization in all ASSAE patients, continuous EEG monitoring was not uniformly available throughout the entire 72-h post-PER assessment window, which may have affected the detection of subclinical seizures.

## 5 Conclusion

In this small retrospective case series, seizure improvement was observed in a subset of pediatric patients with autoimmune encephalitis–related seizures or autoimmune-associated epilepsy after PER administration, and no serious adverse events were recorded. However, because most patients received concomitant ASMs and immunotherapy, the independent contribution of PER cannot be determined. These preliminary findings suggest that PER may be considered as an adjunctive option in selected patients, but larger prospective controlled studies are needed to clarify its efficacy, safety, optimal timing, and patient selection.

## Data Availability

The original contributions presented in the study are included in the article/supplementary material, further inquiries can be directed to the corresponding authors.
